# Studies on the mechanisms of action of MR33317

**DOI:** 10.1007/s00210-024-03226-0

**Published:** 2024-06-10

**Authors:** Joachim Neumann, C. Hesse, S. Yahiaoui, P. Dallemagne, C. Rochais, B. Hofmann, U. Gergs

**Affiliations:** 1 Institute for Pharmacology and Toxicology, Medical Faculty, Martin Luther University Halle-Wittenberg, Magdeburger Straße 4, 06097 Halle (Saale), Germany; 2 Normandie Univ, UNICAEN CERMN (Centre d’Etudes Et de Recherche Sur Le Médicament de Normandie), 14032 Caen, France; 3 Department of Cardiac Surgery, Mid-German Heart Center, University Hospital Halle, Ernst-Grube Straße 40, 06097 Halle (Saale), Germany

**Keywords:** MR33317, 5-HT_4_-receptors, Transgenic mice, Human atrium

## Abstract

MR33317 was synthesized as an acetylcholinesterase-inhibitor and an agonist at brain 5-HT_4_-receptors. MR33317 might be used to treat Morbus Alzheimer. This therapeutic action of MR33317 might be based on MR33317´s dual synergistic activity. We tested the hypothesis that MR33317 also stimulates 5-HT_4_-receptors in the heart. MR33317 (starting at 10 nM) increased force of contraction and beating rate in isolated atrial preparations from mice with cardiac confined overexpression of the human 5-HT_4_-serotonin receptor (5-HT_4_-TG) but was inactive in wild type mouse hearts (WT). Only in the presence of the phosphodiesterase III-inhibitor cilostamide, MR33317 raised force of contraction under isometric conditions in isolated paced (1 Hz) human right atrial preparations (HAP). This increase in force of contraction in human atrium by MR33317 was attenuated by 10 µM tropisetron or GR125487. These data suggest that MR33317 is an agonist at human 5-HT_4_-serotonin receptors in the human atrium. Clinically, one would predict that MR33317 may lead to atrial fibrillation.

## Introduction

Counteracting a deficiency of acetylcholine is an established approach in the drug treatment of Alzheimer's disease. For this reason, cholinesterase inhibitors have entered the clinic in order to control symptoms of Morbus Alzheimer (Briggs et al. 2016). However, drugs with mechanisms of action in addition to inhibition of cholinesterases might offer a more efficient treatment of this disease, at least in some patients. An additional target receptor might be the brain 5-HT_4_-serotonin receptor (Karayol et al. 2021; Reynolds et al. 1995). Indeed, Lecoutey and coworker (2014) have synthesized in donecopride a molecule, that activates the 5-HT_4_-serotonin receptors and acts as a partial agonist with respect to cAMP formation in 5-HT_4C_-serotonin receptor-transfected cells (binding affinity = 10.4 nM). They had developed this compound because studies have shown that agonists at 5-HT_4_-receptors were promising agents to slow the progress or might even be useful to reverse Morbus Alzheimer (Jiang et al. 2024). 5-HT_4_-serotonin receptors can release acetylcholine in neuronal tissue (Kilbinger and Wolf 1992). For that reasoning, Lecoutey et al. (2014) designed in donecopride a drug that is also an acetylcholinesterase inhibitor (IC_50_-value = 16 nM, Lecoutey et al. 2014). Acetylcholinesterases break an ester bond in the molecule acetylcholine, an important neurotransmitter. Acetylcholinesterase inhibitors impair this inactivation of acetylcholine and thus increase brain levels of acetylcholine. One current hypothesis is that when levels of acetylcholine increase in the brain the memory function in the patient improves. Thus, donecopride has, at least, two different mechanisms of action that might improve memory function. Hence, these two mechanisms of actions might both synergistically increase acetylcholine levels in the brain. Finally, 5-HT4-receptors can lead in the brain to activate an enzyme (α-secretase) that may slow the progress of Morbus Alzheimer (Cho and Hu 2007). In some mouse models of Morbus Alzheimer beneficial cognitive effects of donecopride were measured (Lecoutey et al. 2014; Rochais et al. 2020). It remains to be shown whether donecopride or similar compounds would be beneficial in clinical studies against Alzheimer’s disease (Roux et al. 2021).

MR333317 was conceived as another dual compound targeting both acetylcholinesterase and 5-HT_4_- serotonin receptor, with a potential interest towards Alzheimer’s disease. However, MR33317 may have cardiac effects. If MR33317 is taken parenterally or perorally, it has to pass the heart, before MR33317 can enter the brain. Hence, cardiac effects of MR33317 are to be expected especially via human 5-HT_4_-serotonin receptors. It is accepted that all inotropic and chronotropic effects of serotonin are mediated via 5-HT_4_-serotonin receptors on human cardiomyocytes (reviews: Kaumann and Levy 2006; Neumann et al. 2017). In mouse heart, serotonin does not increase force of contraction from wild type mice (WT, Gergs et al. 2010, 2013). Hence, it is impossible to know from studies with wild type mice whether or not MR33317 stimulates cardiac 5-HT_4_-serotonin receptors. To facilitate study of human 5-HT_4_-serotonin receptors, we therefore established in the past a transgenic mouse with overexpression of this receptor (5-HT_4_-TG) only in the heart, which responds with positive inotropic and positive chronotropic effects to agonists (Gergs et al. 2010; review: Neumann et al. 2017).

**Fig. 1:**
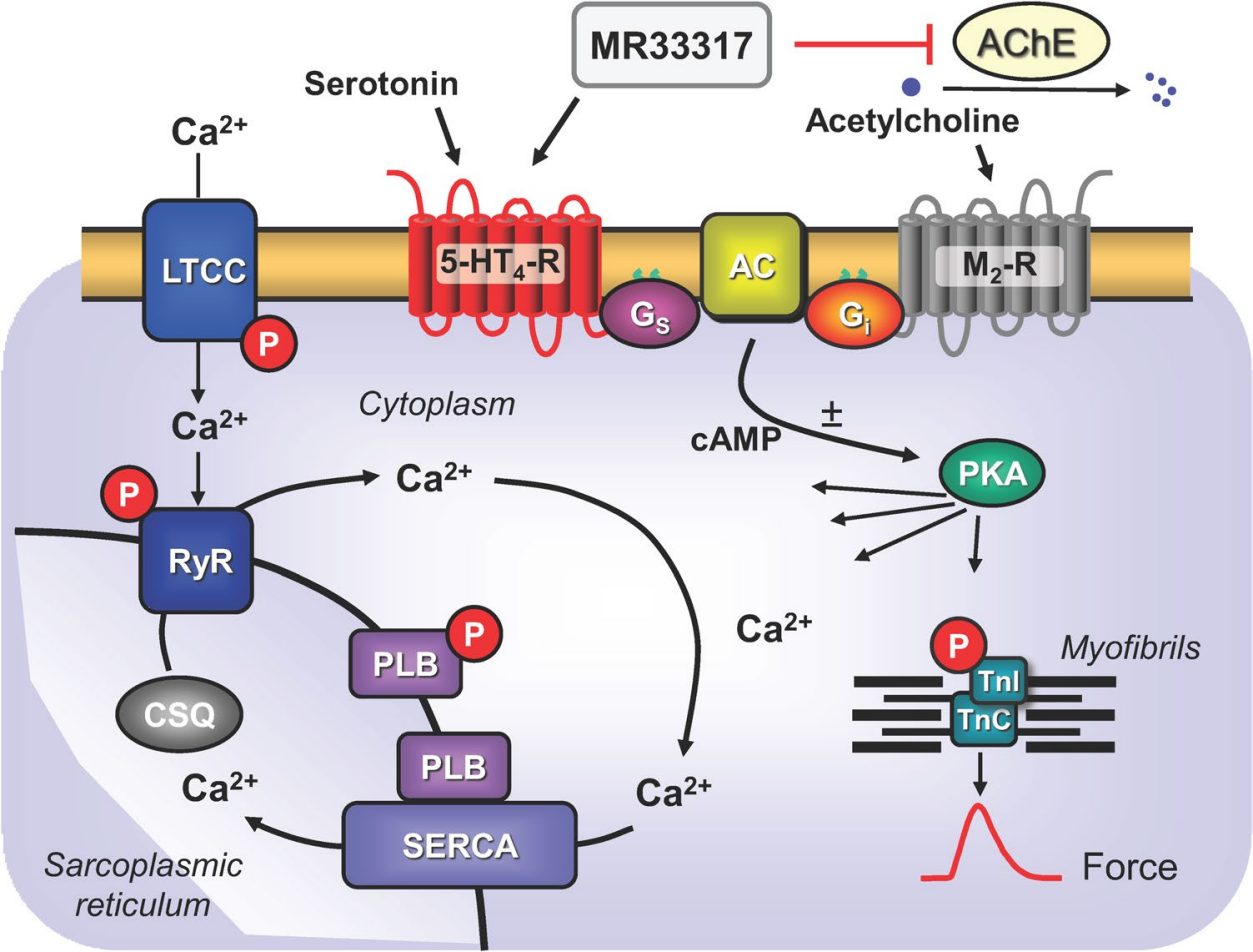
Mechanism(s) of action of MR33317 in human cardiomyocytes: Serotonin stimulates 5-HT_4_-serotonin-receptors in the sarcolemma. These activate stimulatory GTP-binding proteins (Gs). Gs stimulate adenylyl cyclases (AC) that catalyze the formation of cAMP. MR33317 can stimulate these 5-HT_4_-serotonin-receptors. Moreover, MR33317 can inhibit acetylcholinesterases (AChE). Acetylcholine can act on M_2_-muscarinic receptors (M_2_-R). In a next step, a cAMP-dependent protein kinase (PKA). PKA stimulates several regulatory proteins like phospholamban (PLB). PLB, when phosphorylated stimulates the uptake of Ca^2+^ into the sarcoplasmic reticulum. This leads to quicker decrease in Ca^2+^ and thus muscles relax faster

Hence, we decided to test whether MR33317 would exert positive inotropic and positive chronotropic effects in atrial preparations from 5-HT_4_-TG and not in littermate WT. If that were the case, one would expect MR33317 also to stimulate the 5-HT_4_-serotonin receptors in the human heart and thereby increase force of contraction (Fig. 1). We used this reasoning with some success in the past. For instance, we found out that metoclopramide stimulated 5-HT_4_-serotonin receptors in the atrium and ventricle of 5-HT_4_-TG as well as the isolated human atrium. This cardiac effect of metoclopramide is an off-target effect of metoclopramide because the intended use of metoclopramide is in the gastric tract where it stimulates gastric 5-HT_4_-serotonin receptors. In similar way, we have recently noted that some hallucinogenic drugs like psilocin or lysergic acid diethylamide (LSD) can stimulate cardiac 5-HT_4_-serotonin receptors in the 5-HT_4_-TG (Dimov et al. 2023; Jacob et al. 2023). Hence, there is precedence for unintended cardiac effect of drugs with an intended action on other organs (gut, brain) and our approach seems reasonable, though speculative.

Hence, we mainly tested the following hypotheses: MR33317 increases force of contraction firstly in atrial preparations from 5-HT_4_-TG and secondly in human atrial preparations via 5-HT_4_-serotonin receptors.

## Materials and methods

### Contractile studies in mice

Transgenic mice were generated by overexpressing the cDNA of the full length human 5-HT4-serotonin receptor in the heart (Gergs et al. 2010). To this end we used the α-myosin heavy chain promoter which directed the gene of interest to the heart (Gergs et al. 2010). The initial founder was crossed for many generations in a CD-1 background (Gergs et al. 2010). In brief, the right or left atrial preparations from the mice were isolated and mounted in organ baths as previously described (Gergs et al. 2013; Neumann et al. 2003). The bathing solution of the organ baths contained 119.8 mM NaCI, 5.4 mM KCI, 1.8 mM CaCl_2_, 1.05 mM MgCl_2_, 0.42 mM NaH_2_PO_4_, 22.6 mM NaHCO_3_, 0.05 mM Na_2_EDTA, 0.28 mM ascorbic acid and 5.05 mM glucose. The solution was continuously gassed with 95% O_2_ and 5% CO_2_ and maintained at 37°C and pH 7.4 (Neumann et al. 1998, 2003). Spontaneously beating right atrial preparations from mice were used to study any chronotropic effects. Mice were of female sex, with an age range from 98 to 218 days and have an average age of 127 ± 16 days.

### Contractile studies on human preparations

The contractile studies on human preparations were done using the same setup and buffer as used in the mouse studies (see Contractile studies in mice). The samples were obtained from 11 male patients and 4 female patients, with an age range from 52 to 84 years and have an average age of 67 ± 3 years. Drug therapy included acetylsalicylic acid, allopurinol, amlodipine, apixaban, candesartan, citalopram, clopidogrel, dapagliflocine doxazosin, eplerenone, furosemide, hydrochlorothiazide, ivabradine, lercanidipine methylprednisolone, metformin, metoprolol, mirtazapine, moxonidine, nebivolol, pantoprazol, ranitidine, repaglinide, rivaroxaban, sacubitril, sitagliptin, statins, thyroxine, torasemide, valsartan and xipamide. Patients suffered from two to three vessel coronary heart disease, atrial fibrillation, hypertension, treated testicular carcinoma, impaired kidney function, shingles, pulmonary emphysema, gout and anaemia. Our methods used for atrial contraction studies in human samples have been previously published and were not altered in this study (Gergs et al. 2009, 2017, 2018; Boknik et al. 2019).

### Synthesis of compound MR33317 (1-(4-amino-5-chloro-2-methoxyphenyl)-3-(1-(3-methylbenzyl)piperidin-4-yl)propan-1-one)

We have synthesized MR33317 starting from compound A and 3-(bromomethyl)aniline hydrobromide in a similar manner as previously described for analog derivatives (Lecoutey et al 2014).To a solution of 80 mg (0.20 mmol) of compound **A** in DCM (5 mL) (Lecoutey et al 2014) was added TFA (1 mL) dropwise. The reaction mixture was stirred at room temperature for 1 h and then concentrated under reduced pressure. Evaporation of the solvent provided a light yellow oil, which was thereafter dissolved in 1,4-dioxane (30 mL). To the resulting solution, 20 equiv. of K_2_CO_3_ (553 mg, 4.0 mmol) were added and thereafter 1.2 equiv. of 3-(bromomethyl)aniline hydrobromide (64 mg, 0.24 mmol) was added. The reaction mixture was then stirred at reflux until the full consumption of the starting material. The mixture was then concentrated *in vacuo*, diluted with water and extracted twice with EtOAc. The combined organic phases were washed with brine, dried over MgSO_4_, filtrated and concentrated under pressure. The crude product was thereafter purified by silica column flash chromatography using EtOAc–MeOH-Et_3_N (9.75:0.125:0.125 then 9.5:0.25:0.25), giving MR33317 (49 mg) in 61% yield (2 steps) as yellow-pale oil: ^1^H NMR (400 MHz, CDCl_3_): δ 7.76 (s, 1H), 7.08 (t, *J* = 7.6 Hz, 1H), 6.76–6.64 (m, 2H), 6.58 (*br* dd, *J* = 2.4, 8.0 Hz, 1H), 6.24 (s, 1H), 4.52 (s, 2H), 3.81 (s, 3H), 3.43 (s, 2H), 2.99–2.76 (m, 6H), 1.95 (*br* t, *J* = 10.2 Hz. 2H), 1.74–1.62 (m, 2H), 1.57 (*br* q, *J* = 6.8 Hz, 2H), 1.38–1.16 (m, 3H); ^13^C NMR (100 MHz, CDCl_3_): δ 199.4, 159.7, 148.0, 146.6, 138.9, 132.3, 129.2, 120.0, 118.9, 116.2, 114.2, 111.3, 97.6, 63.4, 55.7, 53.8, 40.9, 35.4, 31.9, 31.2; HRMS (*m*/*z*) calcd for C_22_H_29_ClN_3_O_2_ [M + H]^+^ 402.194281, found 402.194006.

### Ligand binding and pharmacological profile

The method was validated from saturation studies: six concentrations of MR33317 were used to give final concentrations of 0.0625–2 nM, and nonspecific binding of MR33317 was defined in the presence of 30 μM serotonin to determine the Kd and the Bmax. For competition studies, 2.5 µg of proteins (5-HT_4_B membrane preparations, HTS110M, Millipore. Millipore’s 5-HT_4_B membrane preparations are crude membrane preparations made from their proprietary stable recombinant cell lines to ensure high-level of GPCR surface expression.) were incubated in duplicate at 25 °C for 60 min in the absence or the presence of 10–6 or 10-8 M of each drug and 0.2 nM MR33317 (VT 240, ViTrax) in 25 mM Tris buffer (pH 7.4, 25 °C). At the end of the incubation, homogenates were filtered through Whatman GF/C filters (Alpha Biotech) presoaked with 0.5% polyethylenimine using a Brandel cell harvester. Filters were subsequently washed three times with 4 mL of ice-cold 25 mM Tris buffer (pH 7.4, 4 °C). Non-specific binding was evaluated in parallel in the presence of 30 µM serotonin. Affinity constants was calculated from five-point inhibition curves using the EBDA-Ligand software and expressed as Ki ± SD. Determination of cAMP production by MR33317 was achieved by Eurofins society.

### Acetylcholinesterase activity

Inhibitory capacity of MR33317 on AChE biological activity was evaluated through the use of the spectrometric method of Ellman (Ellman et al 1961). Acetylthiocholine iodide and 5,5-dithiobis-(2-nitrobenzoic) acid (DTNB) were purchased from Sigma Aldrich. AChE from human erythrocytes (buffered aqueous solution, ≥ 500 units/mg protein (BCA), Sigma Aldrich) was diluted in 20 mM HEPES buffer pH 8, 0.1% Triton X-100 such as to have enzyme solution with 0.25 unit/mL enzyme activity. In the procedure, 100 μL of 0.3 mM DTNB dissolved in phosphate buffer pH 7.4 were added into the 96 wells plate followed by 50 μL of test compound solution and 50 μL of enzyme (0.05 U final). After 5 min of preincubation at 25 °C, the reaction was then initiated by the injection of 50 μL of 10 mM acetylthiocholine iodide solution. The hydrolysis of acetylthiocholine was monitored by the formation of yellow 5-thio-2-nitrobenzoate anion as the result of the reaction of DTNB with thiocholine, released by the enzymatic hydrolysis of acetylthiocholine, at a wavelength of 412 nm using a 96-well microplate plate reader (TECAN Infinite M200, Lyon, France). Test compounds were dissolved in analytical grade DMSO. Donepezil was used as a reference standard. The rate of absorbance increase at 412 nm was followed every minute for 10 min. Assays were performed with a blank containing all components except acetylthiocholine, in order to account for non-enzymatic reaction. The reaction slopes were compared and the percent inhibition due to the presence of test compounds was calculated by the following expression: 100—(vi/v0 × 100) where vi is the rate calculated in the presence of inhibitor and v0 is the enzyme activity.

First screening of AChE activity was carried out at a 10^–6^ or 10^−5^ M concentration of MR33317 under study. IC_50_ values were determined graphically by plotting the % inhibition versus the logarithm of six inhibitor concentrations in the assay solution using the Origin software.

### Data analysis

Data shown are means ± standard error of the mean. Statistical significance was estimated using the analysis of variance followed by Bonferroni’s t-test. A p-value < 0.05 was considered to be significant.

### Drugs and materials

The drugs isoprenaline-hydrochloride, MR33317 10 mM was dissolved in dimethylsulfoxide (DMSO), acetylcholine, rolipram, cilostamide and carbachol (CAR) were purchased Sigma-Aldrich (Germany). All other chemicals were of the highest purity grade commercially available. Deionized water was used throughout the experiments. Stock solutions were prepared fresh daily.

## Results

### Synthesis

**Fig. 2:**
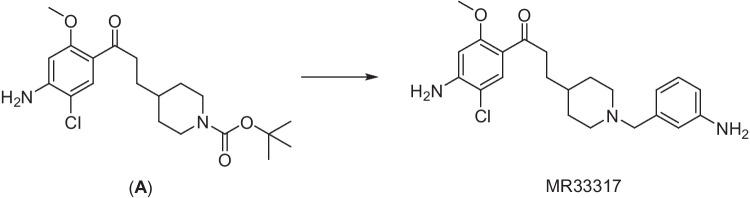
Synthesis of MR33317

Using the reaction depicted in Fig. 2, MR33317 was obtained in 61% yield and high purity (> 98%).

### Binding at 5-HT_4_-receptor

We noticed that MR33317 bound with high affinity to recombinant 5-HT_4_-receptor. The Ki-values of MR33317 amounted to 42.5 ± 2.6 nM (*n* = 3). It acted as a partial agonist at recombinant 5-HT_4_-receptor with 35.6% of the activity of 10 µM serotonin (*n* = 2).

### Acetylcholinesterase inhibition

MR33317 also inhibited in vitro the enzyme activity with an IC_50_-value of 41 ± 5 nM (*n* = 3). 1 µM MR33317 inhibited the activity of acetylcholinesterase by 93% (*n* = 3).

**Fig. 3:**
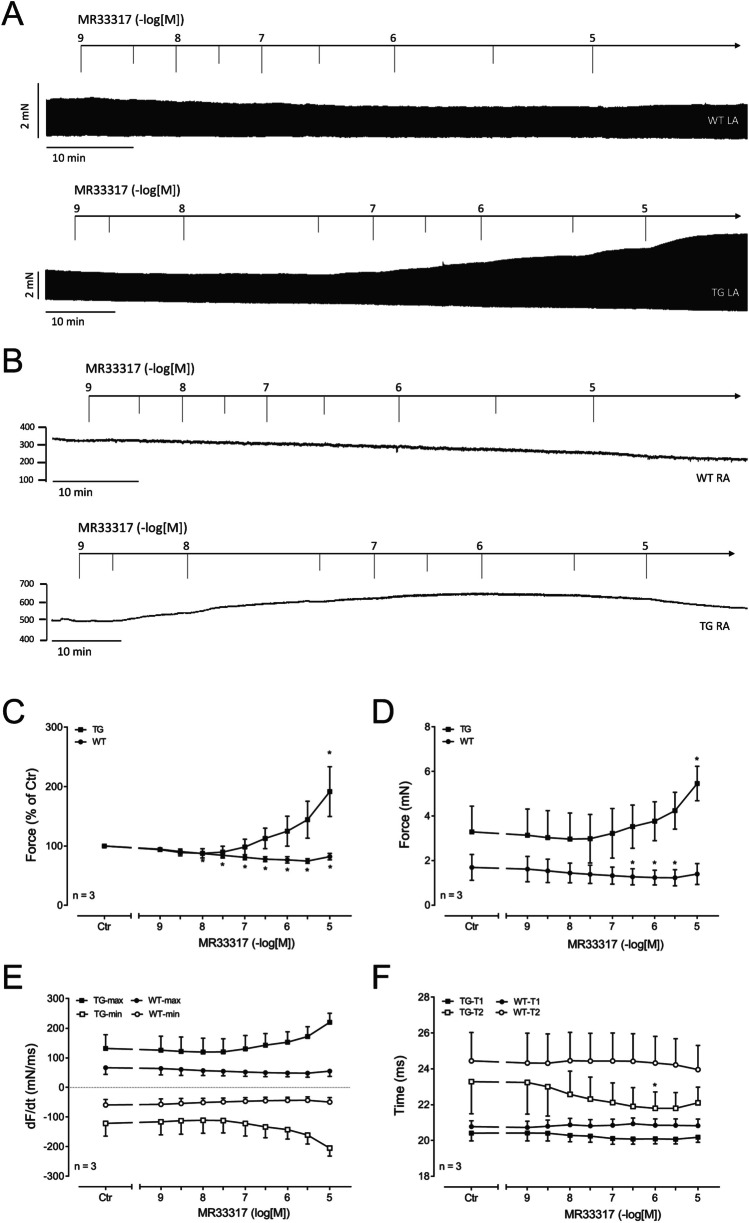
Original recording in mouse left atrial preparation from 5-HT_4_-TG (Fig. 3A, bottom). MR33317 induced a time- and concentration-dependent positive inotropic effect in 5-HT_4_-TG. Original recordings as in WT (Fig. 3A, top). Original recording in mouse right atrial preparation from 5-HT4-TG (Fig. 3B, bottom) and from WT (Fig. 3B, top). Summarized concentration–response curves for the effect of MR33317 on force of contraction (Fig. 3C-D): Force of contraction given as % of Ctr (pre-drug value) or milli Newton (mN) respectively. Figure 3E: rate of tension development (dF/dt_max_) and rate of relaxation (dF/dt_min_) in mN/s. Figure 3F: time to peak tension (T1) and time of relaxation (T2) in milli seconds (ms). Abscissae indicate concentrations of MR33317 in negative decadic logarithm of molar concentrations. *p < 0.05 vs. Ctr; *n* = 3

Contraction studies: MR33317 exerted a concentration- and time-dependent positive inotropic effect in left atrial preparations from 5-HT_4_-TG. This is depicted in an original recording (Fig. 3A, bottom). One can see that the longer MR33317 is in the organ bath, force of contraction increases. In contrast, in left atrial preparations from WT, MR33317 failed to increase force contraction (Fig. 3A, top). This suggests that MR33317 acts only in 5-HT_4_-TG and therefore most probably via 5-HT_4_-receptors. Several such experiments are plotted in Fig. 3.

**Fig. 4:**
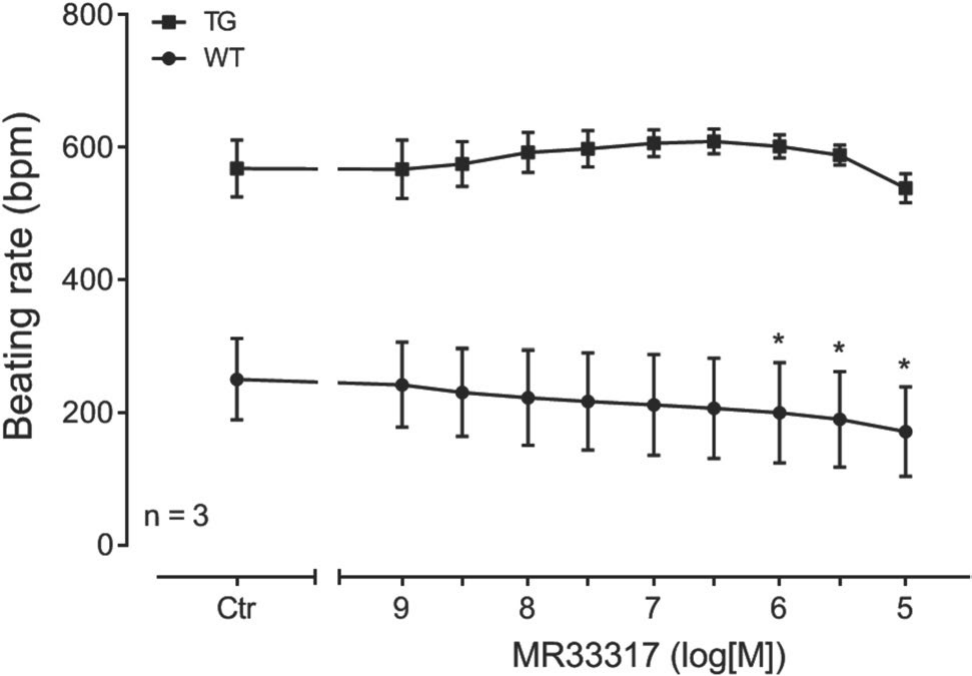
MR33317 induced a time- and concentration-dependent positive chronotropic effect in right atrial preparations from 5-HT_4_-TG but not WT. Summarized concentration–response curve for the effect of MR33317 in right atrium on beating rate. *p < 0.05 vs. Ctr; *n* = 3

If MR33317 behaved like 5-HT, MR33317 should exert effects on the beating rate in right atrium of 5-HT_4_-TG. Indeed, we noticed a time- and concentration-dependent positive chronotropic effect of MR33317 that is plotted in right atrium (Fig. 4). High concentrations of MR33317 led to arrhythmias (Fig. 4).

**Fig. 5:**
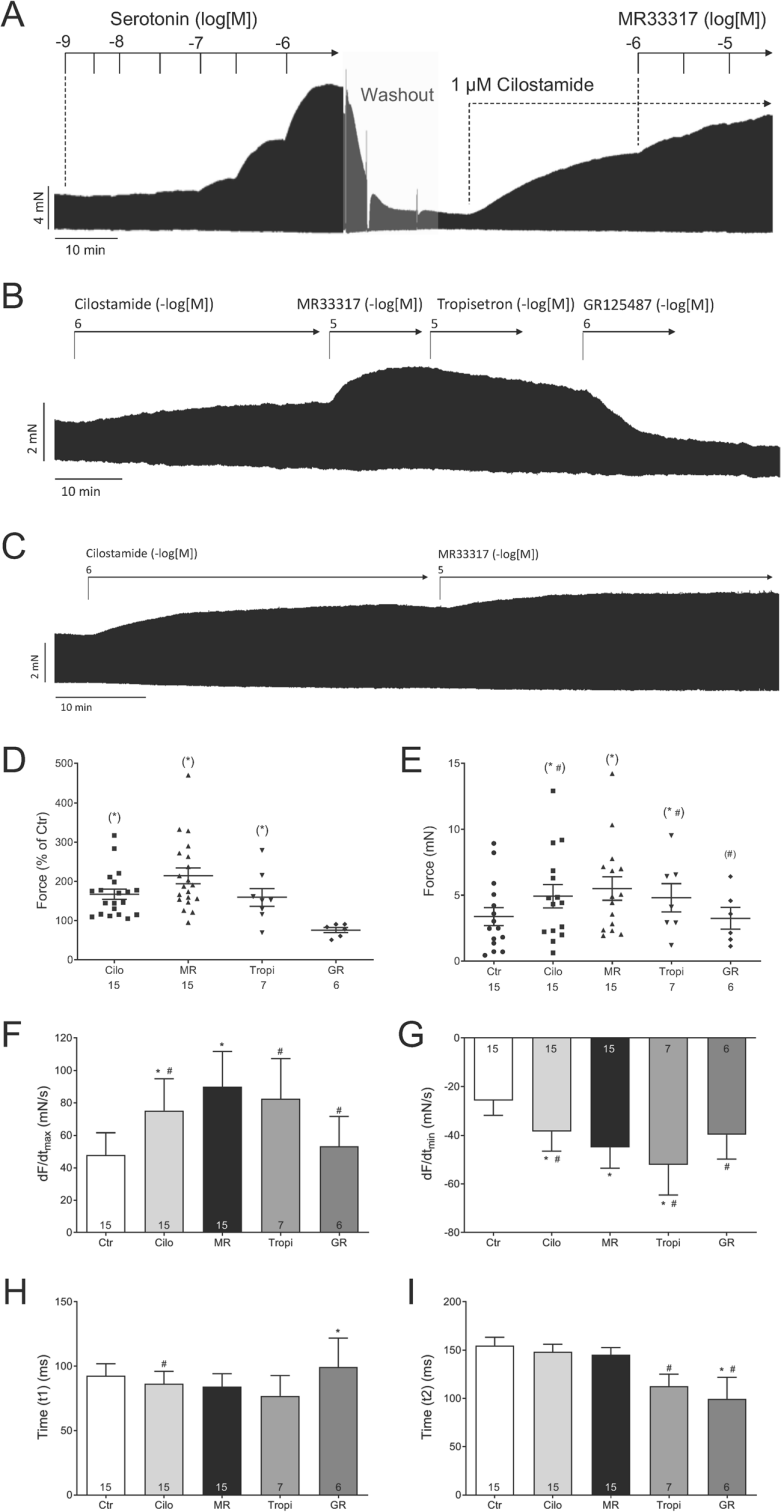
Original recording: First in order show that the muscle is responding in a time and concentration dependent fashion of serotonin, we cumulatively applied serotonin. Serotonin induced a concentration- and time-dependent positive inotropic effect of serotonin measured in milli Newton (mN) in electrically stimulated HAP (Fig. 5A, left hand side). Then after wash out of serotonin and when force fell to low values, cilostamide, a phosphodiesterase III inhibitor was added to pre-stimulated the force of contraction. The effect of cilostamide on force of contraction had reached a plateau thereafter MR33317 was added (Fig. 5A, right hand side). Note that the increase in force is slow under MR33317 compared to serotonin. In a separate experiment, tropisetron to small extend and GR125487 to a full extend, antagonized the effect of MR33317 in the presence of MR33317 on force of contraction in HAP. In a separate experiment (Fig. 5C), no initial serotonin was added only cilostamide and then MR33317. Here (Fig. 5C), MR33317 likewise increased force of contraction but even slower than in Fig. 5A or Fig. 5B. Horizontal bar indicates time axis in minutes (min). Design was subsequently like in Fig. 5B, with the except that only GR125487 was applied. Figure 5D and Fig. 5E: Force of contraction given as % of Ctr (pre-drug value) or milli Newton (mN) respectively in dot plots (indicating individual HAP), Fig. 5F and 5G: rate of tension development and rate of relaxation, Fig. 5H and 5I: time to peak tension (t1) and time of relaxation (t2). Ordinates in Fig. 5A, Fig. 5B, Fig. 5C and Fig. 5E: force of contraction in milli Newton (mN). Figure 5F and 5G in milli Newton per second (mN/s). Figure 5H and 5I in milli seconds (ms). Bars or indicate pre-drug value (Ctr), cilostamide (1 µM Cilo), MR33317 (10 µM MR), tropisetron (10 µM TR) of MR33317 in negative decadic logarithms. Significant differences versus Ctr are marked with asterisks. Numbers in bars or below dot blots mean number of experiments. # indicate significant differences versus MR

Next, we wanted to test the effects of MR33317 in the human heart. To this end, we mounted human atrial preparations in the organ bath under isometric conditions, stimulated them electrically at physiological rate (1 Hz) and tried to obtain concentration–response curves for MR33317. Probably because MR33317 is less effective at 5-HT_4_-receptors in human atria, we failed to register any positive inotropic effects of MR33317 in the human atrium when it was applied alone: force of contraction in HAP with 10 µM MR33317 amounted to 94 ± 11% of pre-drug value (*n* = 9, *p* < 0.05). (data not shown). In another approach (Fig. 5A), we first applied 5-HT cumulatively and raised force of contraction indicating the potency and efficacy of 5-HT in the human atrium. Thereafter, we washed out serotonin and in the very same preparation gave cilostamide to raise force by inhibiting the activity of phosphodiesterase III (Figs. 1A and 5A); then probably cAMP levels were elevated and this led via various phosphorylations of Ca^2+^-regulatory proteins to an increase in force of contraction and a decrease in the time of relaxation (Fig. 1A). When thereafter MR33317 was applied, we detected a positive inotropic effect of MR33317 in a concentration- and time-dependent way (Fig. 5A). MR33317 increased force of contraction (in the presence of cilostamide) and this increase was attenuated in a separate experiment by subsequently applied tropisetron (Fig. 5B). In subsequent experiments we did not give initially serotonin, but simply gave cilostamide and then one concentration (10 µM) of MR33317. This is depicted in a further original recording (Fig. 5C). Such experiments were performed repeatedly and force was evaluated statistically in Fig. 5D and E. Please note that we also have plotted here that subsequently applied tropisetron just barely and GR 125487 much more effectively reduced force of contraction, that had been raised before by MR33317, in the continued presence of cilostamide (Fig. 5D, E). In the same samples, we assessed rates of tension development and rates or relaxation. As seen in Fig. 5F and G, MR3317 in the same HAP increased rate of tension development further from values already elevated by cilostamide (Fig. 5F and G). Similarly, in the same HAP MR33317 decreased rate of tension development further from values already reduced by cilostamide (Fig. 5F and G). While GR125487 prolonged time to peak tension compared to Ctr (pre-cilostamide value) (Fig. 5H, t1), GR125487 shortened time of relaxation (Fig. 5I,t2).

## Discussion

### Mechanism of MR33317

We suggest MR33317 increased force and beating rate as an agonist at cardiac human 5-HT_4_-serotonin receptors. This suggestion is based on the following findings: MR33317 only increased force of contraction in isolated left atrial preparations from 5-HT_4_-TG and not in WT. MR33317 only increased beating rate in isolated right atrial preparations from 5-HT_4_-TG and not in WT. From comparing the concentration- response curves of MR33317 to that of serotonin in atrial preparations (Gergs et al. 2013) one can conclude that MR33317 at 5-HT_4_-serotonin receptors in left and right atrium acts as an agonist.

### Role of phosphorylation of regulatory proteins

The general assumption is that 5-HT_4_-serotonin receptor stimulation leads to an increase in the phosphorylation of proteins that are substrates for the cAMP dependent protein kinase (Fig. 1). We and others described that serotonin via 5-HT_4_-serotonin receptors can increase the phosphorylation state of phospholamban (Gergs et al. 2009; Christ et al. 2014).

Interestingly, MR33317 increased force of contraction via 5-HT_4_-serotonin receptors in the isolated human atrium. We conclude that the 5-HT_4_-serotonin receptors are mediating this effect because two different antagonists namely tropisetron and GR125487 can reduce this effect. This effect did not occur in using MR33317 alone in the human atrium. Only in the presence of a phosphodiesterase we noted a positive inotropic effect. We used cilostamide as a phosphodiesterase inhibitor as before (Jacob et al. 2023). This was done because cilostamide inhibits PDE III and because PDE III is the main PDE in the human heart (Movsesian et al. 2009). In the mouse heart, cilostamide has no positive inotropic effect because PDE IV and not PDE III is abundant in the mouse heart (Neumann et al. 2021b). In the isolated left atrium of wild type mice, the inhibition of PDE IV with rolipram did not unveil a positive inotropic effect of serotonin and therefore one does not expect a positive inotropic effect of MR3317 in the presence of rolipram in the wild type mouse heart under our experimental conditions (Neumann et al. 2019). Moreover, one might conclude that MR33317 acts via cAMP: it is typical that the positive inotropic effects of cAMP increasing agents (e.g. adrenoceptor agonists) are amplified by PDE inhibitors (Feldman et al. 1987). Another argument why one might think that MR33317 acts via cAMP generation is the observation that the MR33317 shortened the time of relaxation in the left atrium of 5-HT_4_-TG. One might ask why MR33317 did not shorten relaxation time in human atrial preparations. One might argue that cilostamide alone reduced relaxation time and conceivably this effect is so large that additionally applied MR33317 cannot shorten time of relaxation any further. This view is supported by the finding that serotonin is less effective than isoprenaline to increase force of contraction in HAP (Kaumann et al. 1990; Gergs et al. 2009). On the other hand, we would point out that the density of 5-HT_4_-serotonin receptors in 5-HT_4_-TG is higher than in HAP. Moreover, the PIE of serotonin was equieffective with isoprenaline to raise force of contraction in left atrial preparations from 5-HT_4_-TG.

Moreover, this paper has the merit that it reports for the first time the synthesis of MR33317. The other novelty is that we report for the first time that MR3317 is a potent inhibitor of the activity in acetylcholinesterases. It might be useful to study in the future whether also cardiac acetylcholinesterases are inhibited in vitro by MR33317. In addition, one might study based on such findings, whether or not MR33317 increases the negative inotropic effect of acetylcholine in the human heart. This is expected from previous reports wherein the negative inotropic or chronotropic effects of acetylcholine in the heart were potentiated by physostigmine, or other acetylcholinesterase inhibitors (Maurer 1979; Korth and Kühlkamp 1987; Kakinuma et al. 2009).

### Species differences

Of note, MR33317 acted more potently and more effectively to raise force in transgenic mice than in human atrium. This is in line with our previous work on cisapride and prucalopride or metoclopramide (Keller et al. 2018; Neumann et al. 2021a). We assume this is due to the much higher level of expression of 5-HT_4_-serotonin receptors in mouse hearts in comparison to human hearts (Neumann et al. 2021a). We would argue that the 5-HT_4_-TG over the possibility of amplifying any effect of agonists at 5-HT_4_-serotonin receptors. One the other hand, if a putative agonist does not act in 5-HT_4_-TG, this agonist is unlikely to work in human tissue as an agonist.

### Effects on beating rate

We assume that like 5-HT also MR33317 stimulated 5-HT_4_-serotonin receptors in the mouse heart. This conclusion is based on the observation that the effect is absent in right atrium from WT The MR33317 acted like various other agonist in our hands (cisapride, prucalopride, metoclopramide, Keller et al. 2018; Neumann et al. 2021a) as a partial agonist compared to the effect of 5-HT on beating rate.

### Clinical relevance

We would predict that a tachycardia after treatment with MR33317 in patients could be blocked by tropisetron, an approved drug. But this prediction needs to be confirmed in a clinical study.

### Limitations of the study

One can argue that we have not tested the effects on the sinus node of man directly. Such a study would require access to the human pacemaker. Moreover, we had a very heterogeneous patient population with respect to heart diseases and comorbidities and past surgeries. Our patients took many potent drugs which might have altered gene expression levels in the heart and thus may have interfered with our findings. We have not studied age dependency of the cardiac effects because of low patient numbers or the cardiac tissue of children. Such studies were beyond the scope of this initial study. We have not shown but only suggest that MR33317 increases phospholamban phosphorylation in the human atrium. We did not have the opportunity to study contractility and phosphorylation in human ventricle tissue for lack of access to that tissue.

In summary, we can now address the hypotheses raised in the Introduction in this way: MR33317 raised force of contraction and beating rate in 5-HT_4_-TG and elevated force of contraction in the human heart via 5-HT_4_-serotonin receptors.

## Data Availability

No datasets were generated or analysed during the current study.
